# Traumatic diaphragmatic injuries in blunt and penetrating trauma in a European trauma centre

**DOI:** 10.1007/s00423-026-04169-1

**Published:** 2026-08-01

**Authors:** Lasse Rehné Jensen, Sarah Andresen, Emma Possfelt-Møller, Søren Steemann Rudolph, Thomas Skaarup Kristensen, Luit Penninga

**Affiliations:** 1 Department of Digestive Diseases, Transplantation and General Surgery, Copenhagen University Hospital - Rigshospitalet, Copenhagen, Denmark; 2 Institute of Clinical Medicine, University of Copenhagen, Copenhagen, Denmark; 3 Department of Anaesthesiology, Center of Head and Orthopaedics, Copenhagen University Hospital - Rigshospitalet, Copenhagen, Denmark; 4 Department of Radiology, Center of Diagnostics, Copenhagen University Hospital - Rigshospitalet, Copenhagen, Denmark; 5 Department of Gastrointestinal Surgery, Aalborg University Hospital, Aalborg, Denmark; 6 Department of Clinical Medicine, Aalborg University, Aalborg, Denmark

**Keywords:** Diaphragm rupture, Diaphragm injuries, Trauma, Blunt trauma, Penetrating injuries

## Abstract

**Purpose:**

Traumatic diaphragmatic injury is an uncommon but clinically important consequence of thoracoabdominal trauma. Diagnosis remains challenging because imaging findings may be subtle. We aimed to describe the clinical characteristics, imaging findings, management, and outcomes of surgically confirmed traumatic diaphragmatic injuries treated at a European trauma centre.

**Methods:**

We conducted a retrospective descriptive case series including all patients with surgically confirmed traumatic diaphragmatic injury who underwent surgery between January 2019 and December 2023. Data included demographics, trauma characteristics, imaging findings, operative management, and postoperative outcomes.

**Results:**

Thirty-nine patients were included (median age 38 [IQR 22–55] years; 87% male); 29 (74%) sustained penetrating trauma and 10 (26%) blunt trauma. The median Injury Severity Score was 19 (IQR 14–31). Preoperative CT was performed in 35 patients (90%), and the diaphragmatic injury was reported on the initial CT in only 60% of these patients. Overall, 23 patients (59%) had extra-abdominal injuries, 12 (31%) experienced major complications (Clavien–Dindo > IIIa), and four (10%) died within 30 days. Patients with blunt trauma generally had greater injury severity, more associated injuries, and poorer outcomes than those with penetrating trauma.

**Conclusion:**

Traumatic diaphragmatic injury remains a challenging diagnosis. In this descriptive case series, the diaphragmatic injury was reported on the initial CT in only 60% of patients who underwent preoperative CT, highlighting the importance of maintaining a high index of suspicion and systematically evaluating the diaphragm during trauma CT interpretation. Larger prospective studies are needed to better define the diagnostic performance of modern CT and optimize management.

## Introduction

Traumatic diaphragmatic injuries have a reported incidence of 0.8%–15% [1–3]. Concomitant injuries are common, particularly in patients with blunt trauma and thoracoabdominal injuries. Overall mortality among patients with diaphragmatic injury ranges from 4% to 41% [2, 4]. However, most studies report mortality rates between 10% and 30%, generally higher in patients with blunt trauma [5–8].

Although diaphragmatic injury can be obvious (e.g., herniation of abdominal contents on chest radiograph or CT), the injury may also be subtle, and imaging studies may be nondiagnostic. Reported CT sensitivity for diaphragmatic injury varies widely in the literature, ranging from 40% to 91% [2, 4, 6, 9, 10]. A high index of suspicion must therefore be maintained, as delayed diagnosis is associated with an increased risk of herniation and strangulation of abdominal organs, which may be life-threatening, with reported mortality of up to 66% [4]. In patients in whom the diagnosis remains uncertain, diagnostic laparoscopy, thoracoscopy, or laparotomy may be required to establish the diagnosis. Diaphragmatic injuries are repaired using open or minimally invasive techniques, and the choice and timing of repair depend on the presence of associated injuries and the patient’s overall condition.

In this study, we describe the clinical characteristics, imaging findings, management, and outcomes of patients with surgically confirmed traumatic diaphragmatic injury treated at a Scandinavian trauma centre. The aim of this study was to describe the clinical characteristics, initial CT detection of diaphragmatic injury, management, and outcomes in this cohort and to present these findings according to trauma mechanism (blunt or penetrating).

## Methods

The study followed the Strengthening the Reporting of Observational Studies in Epidemiology (STROBE) guidelines for reporting observational research. We performed a retrospective descriptive case series based on prospectively registered data, including all consecutive patients with surgically confirmed traumatic diaphragmatic injury admitted to the trauma centre at our hospital between January 1, 2019, and December 31, 2023. Both patients admitted primarily to our institution and those transferred from other hospitals were included.

Data were obtained from the hospital’s Trauma Registry and the EPIC electronic health record system used in the Capital Region of Denmark. Patients without operative confirmation of traumatic diaphragmatic injury were excluded, including those with only a suspected diaphragmatic injury on computed tomography (CT) and those who died before operative confirmation.

Extracted baseline variables included age, sex, American Society of Anesthesiologists physical status classification (ASA), Eastern Cooperative Oncology Group Performance Status (ECOG PS), smoking status, height, weight, and body mass index (BMI). In-hospital variables included systolic blood pressure and heart rate on arrival, blood transfusions, trauma mechanism (blunt or penetrating), initial CT findings, time to diagnosis, concomitant injuries, surgical treatment, Abbreviated Injury Scale (AIS), Injury Severity Score (ISS), American Association for the Surgery of Trauma (AAST) diaphragm injury grade, location and side of diaphragmatic injury, length of stay (LOS) in the intensive care unit and hospital ward, and postoperative complications graded according to the Clavien–Dindo classification. Major complications were defined as Clavien–Dindo grade > IIIa. Time to diagnosis was defined as the time from admission to the first report of diaphragmatic injury on CT or confirmation at surgery, expressed in hours.

At our institution, trauma CT is routinely evaluated in axial, coronal, and sagittal planes. Three-dimensional reconstructions may be performed selectively but are not part of the standard assessment.

The study was designed to provide a descriptive characterization of patients with surgically confirmed traumatic diaphragmatic injury. Outcomes of interest included 30-day mortality, major postoperative complications, length of stay in the intensive care unit and hospital, the proportion of injuries reported on the initial CT examination, time to diagnosis, surgical management, and associated injuries. Findings are presented overall and stratified according to trauma mechanism (blunt or penetrating).

### Statistical analyses

Statistical analyses were performed using IBM SPSS Statistics version 27. Continuous variables are presented as mean ± standard deviation (SD) or median with interquartile range (IQR), as appropriate, and categorical variables as counts and percentages. Because of the small sample size and the highly selected cohort of patients with surgically confirmed traumatic diaphragmatic injury, the study was designed as a descriptive case series. Data are presented descriptively overall and stratified by trauma mechanism (blunt and penetrating). No formal hypothesis testing or multivariable analyses were performed.

## Results

A total of 39 patients with surgically confirmed traumatic diaphragmatic injury were included during the study period. During the same period, approximately 4,500 trauma patients were admitted to the trauma centre, including approximately 1,500 patients with an Injury Severity Score (ISS) > 15. Of the 39 included patients, 10 (26%) sustained blunt trauma, primarily high-velocity motor vehicle accidents, whereas 29 (74%) sustained penetrating trauma, predominantly stab wounds, including one gunshot injury.

Baseline characteristics are presented in Table 1. The median age was 38 years (IQR 22–55), and 34 patients (87%) were male. The median ISS was 19 (IQR 14–31). Most patients had an ASA physical status of I (49%) or II (28%), and an ECOG Performance Status of 0 (69%). Most diaphragmatic injuries were AAST grade III (79%). Patients with blunt trauma tended to be older and had higher ISS values than patients with penetrating trauma. Left-sided injuries were observed in 27 patients (69%), right-sided injuries in 11 (28%), and one patient (3%) had a bilateral injury. Sixteen patients (41%) were haemodynamically unstable on arrival. The median red blood cell transfusion requirement was 2 units (IQR 0–6.5), with higher transfusion requirements observed after blunt trauma.

**Table 1:** Baseline characteristics of patients with traumatic diaphragmatic injury overall and stratified by trauma mechanism

Factors	Blunt trauma (*n* = 10)	Penetrating trauma (*n* = 29)	All (*N* = 39)
Age, years, median (IQR)	52 (43–58)	33 (20–49)	38 (22–55)
Male sex, n (%)	6 (60)	28 (97)	34 (87)
BMI (kg/m²), mean ± SD	32.3 ± 10.7	25.0 ± 4.6	26.7 ± 7.1
ASA, n (%) I II III	3 (30) 6 (60) 1 (10)	16 (55) 5 (17) 8 (28)	19 (49) 11 (28) 9 (23)
ECOG PS, n (%) 0 1 2	5 (50) 4 (40) 1 (10)	22 (76) 6 (21) 1 (3)	27 (69) 10 (26) 2 (5)
Current smoking, n (%)	2 (20)	15 (52)	17 (44)
ISS, median (IQR)	41 (30–49)	18 (14–22)	19 (14–31)
AAST grade, n (%) II III IV	0 (0) 6 (60) 3 (30)	4 (14) 25 (86) 0 (0)	4 (10) 31 (79) 3 (8)
Left-sided injury, n (%)	7 (70)	20 (69)	27 (69)
Right-sided injury, n (%)	3 (30)	8 (28)	11 (28)
Bilateral injury, n (%)	0 (0)	1 (3)	1 (3)
Unstable on arrival, n (%)	7 (70)	9 (31)	16 (41)
Red blood cell transfusion, units, median (IQR)	5 (1–17)	1 (0–4)	2 (0–6.5)
Preoperative CT performed, n (%)	8 (80)	27 (93)	35 (90)
Diaphragmatic injury reported on initial CT, n/N (%)	6/8 (75)	15/27 (56)	21/35 (60)
Time to diagnosis, hours, median (IQR)	1 (1–1.5)	1 (1–2)	1 (1–2)

AAST grade was unavailable for one patient. BMI and smoking status were available for 34 and 37 patients, respectively

AAST, American Association for the Surgery of Trauma; ASA, American Society of Anesthesiologists physical status classification; BMI, body mass index; CT, computed tomography; ECOG PS, Eastern Cooperative Oncology Group Performance Status; IQR, interquartile range; ISS, Injury Severity Score; SD, standard deviation

Preoperative CT was performed in 35 of 39 patients (90%). Among these patients, diaphragmatic injury was reported on the initial CT examination in 21 of 35 patients (60%), including 6 of 8 patients (75%) with blunt trauma and 15 of 27 patients (56%) with penetrating trauma. The median time to diagnosis was 1 h (IQR 1–2) in both trauma groups. One patient experienced a substantially delayed diagnosis because the diaphragmatic injury was initially overlooked in the presence of multiple concomitant injuries.

Treatment and outcomes are summarized in Table 2. Extra-abdominal injuries were present in 23 patients (59%), including all 10 patients with blunt trauma. Major postoperative complications (Clavien–Dindo grade > IIIa) occurred in 12 patients (31%), including 8 of 10 patients (80%) with blunt trauma and 4 of 29 patients (14%) with penetrating trauma. Four patients (10%) died within 30 days, all following blunt trauma.

**Table 2:** Treatment and outcomes of patients with traumatic diaphragmatic injury stratified by trauma mechanism (blunt vs. penetrating)

Factors	Blunt trauma (*n* = 10)	Penetrating trauma (*n* = 29)	All (*N* = 39)
Extra-abdominal injury, n (%)	10 (100)	13 (45)	23 (59)
ICU length of stay, days, median (IQR)	8.5 (0.25–31)	1 (0–1)	1 (0–3)
Hospital length of stay, days, median (IQR)	25 (1–83)	6 (5–11)	6 (5–15)
Major complications (Clavien–Dindo > IIIa), n (%)	8 (80)	4 (14)	12 (31)
30-day mortality, n (%)	4 (40)	0 (0)	4 (10)
Surgical approach, n (%)			
Laparotomy alone	8 (80)	26 (90)	34 (87)
Laparotomy and thoracotomy	2 (20)	1 (3)	3 (8)
Thoracotomy alone	0 (0)	1 (3)	1 (3)
Laparoscopic surgery alone	0 (0)	1 (3)	1 (3)
Diaphragmatic repair	8 (80)	29 (100)	37 (95)

ICU, intensive care unit; IQR, interquartile range

Laparotomy alone was the most common surgical approach and was performed in 34 patients (87%). A combined laparotomy and thoracotomy was performed in three patients (8%), whereas one patient (3%) underwent thoracotomy alone and one patient (3%) underwent laparoscopic surgery alone. Diaphragmatic repair was performed in 37 patients (95%). The two patients who did not undergo repair died during emergency surgery shortly after arrival. The median ICU length of stay was 1 day (IQR 0–3), and the median hospital length of stay was 6 days (IQR 5–15). Patients with blunt trauma generally had longer ICU and hospital stays than those with penetrating trauma.

Table 3 summarizes the distribution of postoperative complications according to the Clavien–Dindo classification. Patients with blunt trauma experienced a greater proportion of high-grade complications, whereas 14 of 29 patients (48%) with penetrating trauma had no postoperative complications. All four grade V complications occurred after blunt trauma.

**Table 3:** Distribution of postoperative complications according to the Clavien–Dindo classification, stratified by trauma mechanism

Clavien–Dindo Grade	Blunt trauma (*n* = 10)	Penetrating trauma (*n* = 29)	All (*N* = 39)
0	0 (0)	14 (48)	14 (36)
I	0 (0)	3 (10)	3 (8)
II	2 (20)	3 (10)	5 (13)
IIIa	0 (0)	5 (17)	5 (13)
IIIb	1 (10)	3 (10)	4 (10)
IVa	2 (20)	1 (3)	3 (8)
IVb	1 (10)	0 (0)	1 (3)
V	4 (40)	0 (0)	4 (10)

Concomitant injuries are summarized in Table 4. Hemopneumothorax was present in 29 patients (74%), liver injury in 20 (51%), splenic injury in 11 (28%), and gastrointestinal injury in 11 (28%). Patients with blunt trauma more commonly sustained rib fractures, pelvic fractures, extremity injuries, head injuries, and kidney injuries, reflecting the greater overall injury burden in this group.

**Table 4:** Concomitant injuries in patients with traumatic diaphragmatic injury

Injury	Blunt trauma (*n* = 10)	Penetrating trauma (*n* = 29)	All (*N* = 39)
Liver	6 (60)	14 (48)	20 (51)
Spleen	2 (20)	9 (31)	11 (28)
Pancreas	1 (10)	1 (3)	2 (5)
Gastrointestinal	3 (30)	8 (28)	11 (28)
Hemopneumothorax	7 (70)	22 (76)	29 (74)
Rib fractures	7 (70)	5 (17)	12 (31)
Pelvic fracture	5 (50)	0 (0)	5 (13)
Extremity fracture/injury	8 (80)	8 (28)	16 (41)
Head	6 (60)	0 (0)	6 (15)
Kidney	2 (20)	0 (0)	2 (5)
Pericardium	1 (10)	3 (10)	4 (10)

## Discussion

We present a descriptive case series of patients with surgically confirmed traumatic diaphragmatic injuries treated at a European trauma centre. Patients with blunt trauma generally had higher injury severity scores, higher AAST diaphragm injury grades, more concomitant injuries, and a greater burden of major complications and 30-day mortality than patients with penetrating trauma. These findings should be interpreted in the context of the substantially greater overall injury burden among patients with blunt trauma and the descriptive nature of this study. No cases of failure of diaphragmatic repair were observed.

The characteristics of our cohort are broadly consistent with previous reports regarding the predominance of penetrating injuries, the distribution of left- and right-sided diaphragmatic injuries, and the observation that blunt traumatic diaphragmatic injury is frequently associated with more severe concomitant injuries and poorer outcomes [2–4, 7–9]. Although higher mortality after blunt trauma has also been reported by others [4, 7, 8], our study was not designed or powered to determine whether trauma mechanism independently influenced mortality.

CT is part of the standard evaluation of patients with high-energy blunt trauma and penetrating thoracoabdominal injuries in many trauma systems. In this descriptive series of surgically confirmed traumatic diaphragmatic injuries, the diaphragmatic injury was reported on the initial CT examination in 21 of 35 patients (60%) who underwent preoperative CT. This finding should not be interpreted as a measure of CT sensitivity, as only patients with surgically confirmed injuries were included and patients without operative confirmation were not evaluated.

Previous studies have reported CT sensitivities ranging from 40% to 91%, with generally lower sensitivity for penetrating than blunt injuries [2, 4, 6, 9–11]. For example, Leung et al. reported an overall sensitivity of 66.7%, including 33.3% for penetrating trauma and 86.7% for blunt trauma [11]. Although our study cannot estimate diagnostic accuracy, the relatively frequent absence of a reported diaphragmatic injury on the initial CT examination highlights the continuing diagnostic challenge of these injuries. This may contribute to delayed or missed diagnosis, particularly as non-operative management of thoracoabdominal injuries becomes increasingly common.

**Fig. 1:**
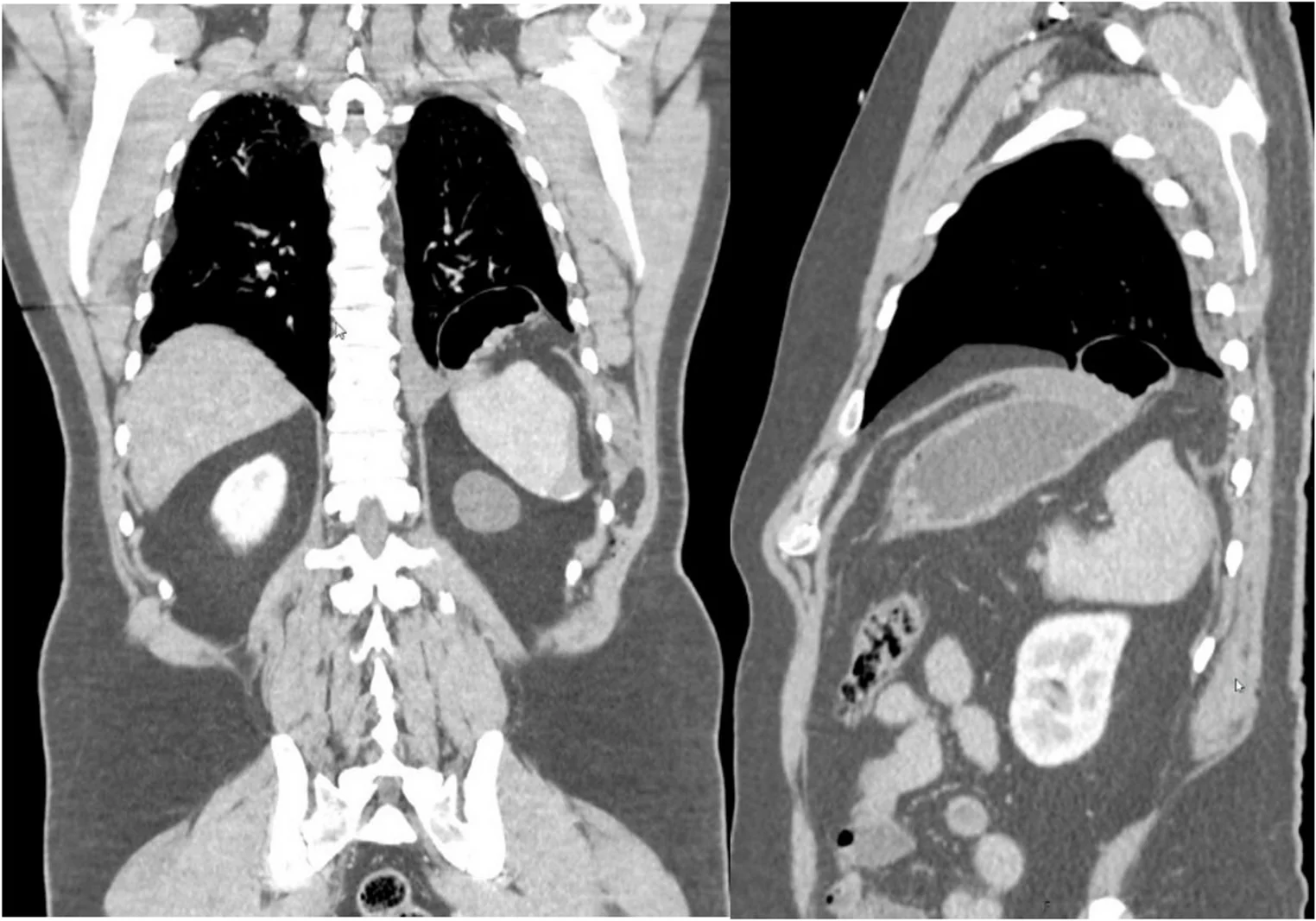
Coronal and sagittal CT images from a patient with blunt trauma. The diaphragmatic injury was identified on the initial CT. Abdominal contents, including the stomach and omentum, are partially herniated into the thorax, and a clear discontinuity of the diaphragm is visible

**Fig. 2:**
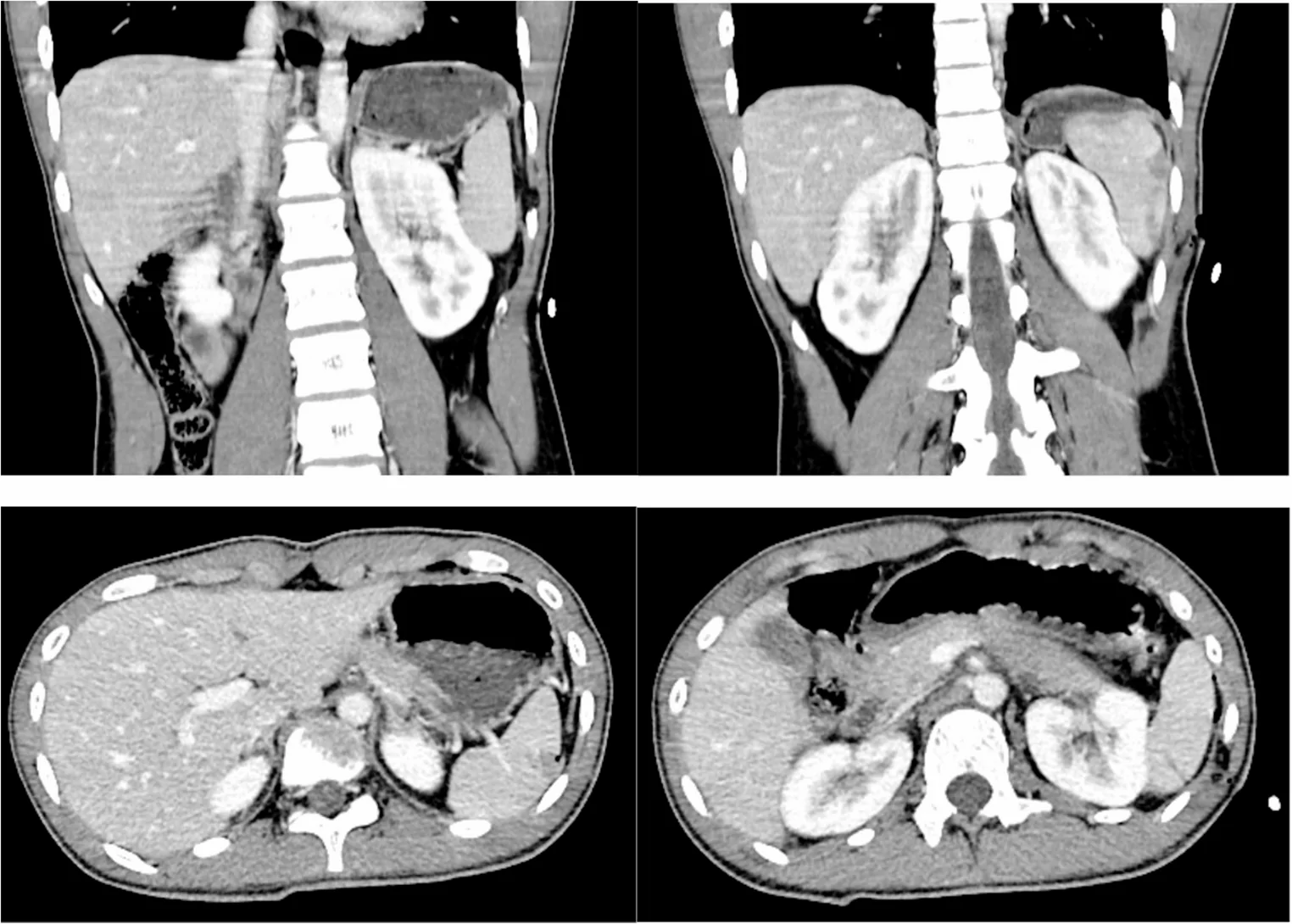
Coronal (**a**, **b**) and axial (**c**, **d**) CT images from a patient with left-sided penetrating trauma. The diaphragmatic injury was not identified on the initial CT interpretation. Panels (**a**) and (**c**) show a subtle discontinuity of the diaphragm, while panels (**b**) and (**d**) demonstrate contiguous injuries on both sides of the diaphragm involving the spleen and subcutaneous tissue

**Fig. 3:**
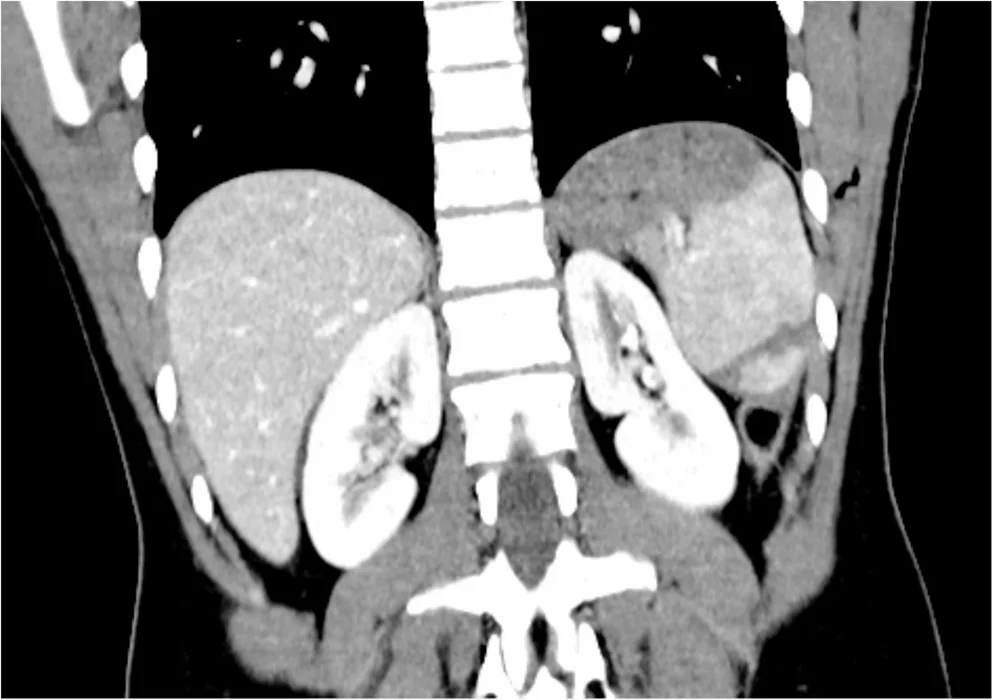
Coronal CT image from a patient with left-sided penetrating trauma. The diaphragmatic injury was not identified on the initial CT interpretation. Injuries to the spleen and subcutaneous tissue are visible at different levels due to diaphragmatic motion during CT acquisition. This represents a subtle example of contiguous injuries on both sides of the diaphragm indicating diaphragmatic injury, which can easily be overlooked if not specifically evaluated

A retrospective review of CT scans from patients in whom the diaphragmatic injury was not reported on the initial CT interpretation showed that indirect signs of diaphragmatic injury were present in several cases but had not been recognized at the time of initial reporting. These included contiguous injuries on both sides of the diaphragm. Examples of CT scans with and without an initially reported diaphragmatic injury are shown in Figs. 1, 2 and 3. Similar observations have been reported following retrospective re-evaluation of trauma CT scans and emphasize the importance of systematic assessment of the diaphragm during interpretation of trauma CT scans [4, 11]. After completion of the study period, newer CT scanners capable of correcting respiratory motion artefacts were introduced at our institution. Whether these technological advances improve the detection of traumatic diaphragmatic injuries remains to be determined. Although our study was not designed to evaluate diagnostic accuracy, our findings highlight the importance of maintaining a high index of suspicion for diaphragmatic injury in patients with thoracoabdominal trauma. If the diagnosis is not considered by either the radiologist or the trauma surgeon, subtle imaging findings may be overlooked. This may be particularly relevant in patients without major associated intra-abdominal injuries, who are increasingly managed non-operatively. In selected patients with persistent clinical suspicion despite inconclusive imaging, diagnostic laparoscopy or thoracoscopy should be considered to establish the diagnosis and allow timely repair before delayed complications, such as visceral herniation or strangulation, develop.

The main strength of this study is the inclusion of a consecutive series of surgically confirmed traumatic diaphragmatic injuries treated at a high-volume European trauma centre, providing detailed clinical, radiological, operative, and outcome data. Nevertheless, several limitations should be acknowledged. First, the sample size was limited, particularly for blunt trauma, and only four deaths occurred. Consequently, the study was not powered to evaluate predictors of mortality or to perform meaningful multivariable analyses. Second, only patients with surgically confirmed diaphragmatic injuries were included. Patients managed non-operatively and those who died before operative confirmation were not captured, introducing selection bias and precluding reliable estimation of the true incidence of traumatic diaphragmatic injury or the diagnostic performance of CT. Third, the retrospective analysis of prospectively collected data is subject to the inherent limitations of retrospective observational studies. Fourth, interpretation of trauma CT scans was performed by different radiologists, introducing potential variability in reporting. Although the institutional CT protocol includes systematic evaluation of axial, coronal, and sagittal reconstructions, diaphragmatic injuries may be subtle and easily overlooked, particularly on axial images. Finally, we did not have access to post-mortem findings from patients who died before operative confirmation, and additional diaphragmatic injuries may therefore have remained unrecognized.

It remains uncertain how many traumatic diaphragmatic injuries are missed in patients managed non-operatively or in those who die before definitive diagnostic evaluation or operative exploration. Consequently, the true incidence of traumatic diaphragmatic injury remains difficult to establish. In a nationwide Japanese study, 25% of patients with traumatic diaphragmatic injury arrived at the hospital in cardiac arrest, illustrating that early mortality may contribute to underestimation of the true disease burden [12]. Further prospective studies including both operative and non-operative patients are needed to better define the diagnostic performance of modern imaging, the incidence of missed injuries, and long-term outcomes, including patient-reported quality of life, pulmonary function, and delayed complications following traumatic diaphragmatic injury.

## Conclusion

Traumatic diaphragmatic injury remains a challenging diagnosis in trauma care. In this descriptive case series of surgically confirmed injuries, just over half of the diaphragmatic injuries were reported on the initial CT examination, underscoring the importance of maintaining a high index of suspicion and systematically evaluating the diaphragm during interpretation of trauma CT scans. Patients with blunt trauma generally sustained more severe associated injuries and experienced poorer outcomes than those with penetrating trauma, although these findings should be interpreted in the context of the greater overall injury burden. Larger prospective studies are needed to better define the diagnostic performance of modern CT and the optimal management of traumatic diaphragmatic injury.

## Data Availability

Data are available from the corresponding author upon reasonable request.
